# Nicotinamide *N*-Methyltransferase: An Emerging Protagonist in Cancer Macro(r)evolution

**DOI:** 10.3390/biom11101418

**Published:** 2021-09-28

**Authors:** Richard B. Parsons, Paul D. Facey

**Affiliations:** 1 Institute of Pharmaceutical Science, King’s College London, 150 Stamford Street, London SE1 9NH, UK; 2 Singleton Park Campus, Swansea University Medical School, Swansea University, Swansea SA2 8PP, UK; p.facey@swansea.ac.uk

**Keywords:** oncogenesis, methylation, tumorigenesis, drug resistance, cancer stem cell, Warburg effect, transcription factors, NAD+

## Abstract

Nicotinamide *N*-methyltransferase (NNMT) has progressed from being considered merely a Phase II metabolic enzyme to one with a central role in cell function and energy metabolism. Over the last three decades, a significant body of evidence has accumulated which clearly demonstrates a central role for NNMT in cancer survival, metastasis, and drug resistance. In this review, we discuss the evidence supporting a role for NNMT in the progression of the cancer phenotype and how it achieves this by driving the activity of pro-oncogenic NAD+-consuming enzymes. We also describe how increased NNMT activity supports the Warburg effect and how it promotes oncogenic changes in gene expression. We discuss the regulation of NNMT activity in cancer cells by both post-translational modification of the enzyme and transcription factor binding to the NNMT gene, and describe for the first time three long non-coding RNAs which may play a role in the regulation of NNMT transcription. We complete the review by discussing the development of novel anti-cancer therapeutics which target NNMT and provide insight into how NNMT-based therapies may be best employed clinically.

## 1. Introduction

**Figure 1:**
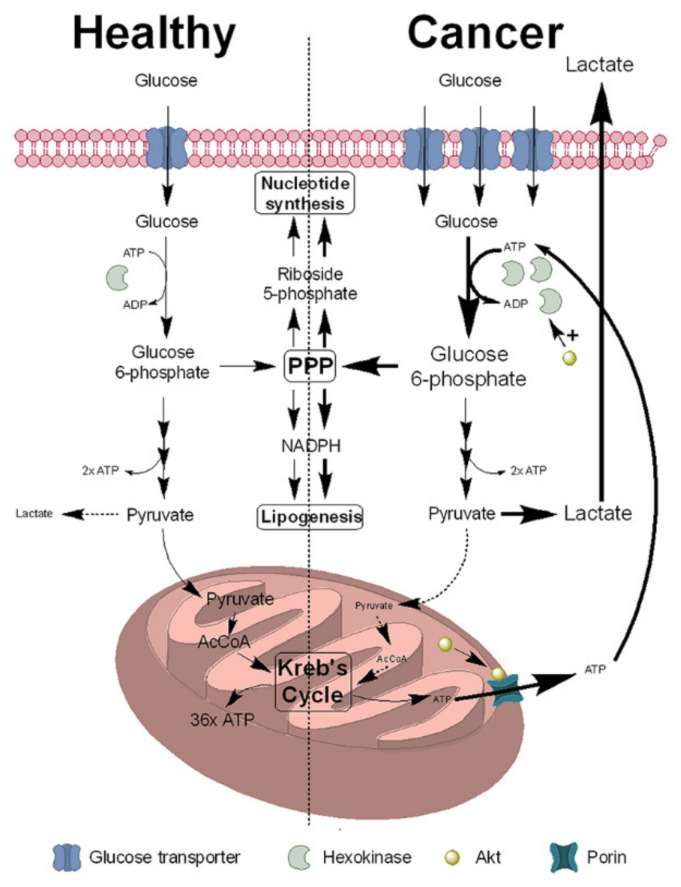
Summary of the metabolic changes within tumour cells arising from the Warburg effect. In healthy cells, glucose is taken up and converted into ATP via oxidative phosphorylation. In the tumour cell, mitochondrial oxidation of pyruvate via the Krebs cycle and the mitochondrial respiratory chain is reduced, mediated by increased expression of lactate dehydrogenase A and pyruvate kinase M2 which shuttles pyruvate into lactate production. The loss in ATP synthesis is compensated by increased expression of the glucose transporter and hexokinase expression, which is induced by Akt signalling. The increase in hexokinase activity is supported by the diversion of mitochondrial ATP to hexokinase, mediated by the binding of Akt to mitochondrial porin. Intermediate products of glycolysis are diverted into the pentose phosphate pathway (PPP), which generates the riboside 5-phosphate and NADPH arising from the increased demand for nucleotide synthesis and lipogenesis arising from the increasing tumour mass. Excess lactate is excreted from the cell, acidifying the extracellular environment, and thus promoting the tumour phenotype over non-tumour cells.

The ability of any mammalian cell to survive relies upon its need to convert fuel, most commonly glucose, into ATP energy. Non-neoplastic tissues do this via the process of oxidative phosphorylation, comprising glycolysis, the Krebs cycle, and the mitochondrial respiratory chain. In contrast, in neoplastic cells, energy metabolism undergoes a metabolic shift, called the Warburg effect, whereby cells rely predominantly upon glycolysis for their energy needs (for a full in-depth review of the Warburg effect in cancer, see [1]). This change in ATP supply source reduces the number of ATP molecules produced per glucose molecule from a (theoretical) maximum of 38 to 2 [2]; such a marked decrease seems counterproductive to cell survival at first glance. However, this shift in metabolism is essential for tumour growth and survival as it provides the necessary building blocks for tumour mass production [3,4] as well as generating a cellular microenvironment which promotes tumour growth over that of surrounding non-neoplastic tissues [5,6,7]. Pyruvate is diverted from entry into the mitochondria and towards the formation of lactate due to increased lactate dehydrogenase A and pyruvate kinase M2 expressions [8,9]. Additionally, pyruvate dehydrogenase kinase 1 activity is increased, which in turn phosphorylates, and thus inhibits the activity of, pyruvate dehydrogenase, the rate-limiting step in the conversion of pyruvate into acetyl-CoA [1]. The subsequent increase in cellular lactate is excreted out of the cell. Accumulating glycolysis intermediates are diverted into the pentose phosphate pathway (PPP) to synthesise the biochemical components required for tumour growth, such as nucleotides and lipids [10,11]. A small proportion of pyruvate enters oxidative phosphorylation, which is essential for regenerating the NAD(P)H used by these pathways, hence mitochondrial activity slows but does not stop (Figure 1) [12,13]. The decrease in ATP production which arises because of the Warburg effect is thought to be compensated for in part by an increase in the rate of glycolysis [14,15], fuelled by increased expression of the glucose transporter and subsequent increase in glucose uptake [16]. Additionally, glycolytic ATP synthesis is approximately 100-fold more rapid than mitochondrial ATP synthesis [17], thus cellular ATP can be maintained at levels similar to those observed in healthy cells [18].

The Warburg effect is not the only change within the neoplastic cell to occur. Tumour cells are characterised by changes in epigenetic regulation, decreased apoptosis, alterations in cellular signalling, promotion of metastasis and development of drug resistance [19]. How to tie all these seemingly disparate processes together is still unclear. In this review, we introduce a new player in the evolution of the cancer phenotype—nicotinamide *N*-methyltransferase (NNMT)—and discuss the evidence which places NNMT in the centre of a web of pathways which promotes the metabolic and cellular changes observed in many cancer cells. We will also discuss the challenges faced in designing cancer therapeutics based upon NNMT and show that NNMT-based therapies are potentially a macrorevolution in cancer treatment.

## 2. NNMT Expression Is Increased in Cancer Cells to Support the Warburg Effect

### 2.1. NAD+ Synthesis

NAD+ is essential for the survival of the cell. In the form of NADH, it is required for the donation of protons and electrons to the mitochondrial respiratory chain for the generation of ATP [20]. NAD+ is also a substrate for several proteins, for example PARP1, which repairs single-strand DNA breaks [21], and sirtuins, which play a role in epigenetic regulation and energy metabolism [22,23]. Cleavage of NAD+ by NAD+-dependent enzymes releases nicotinamide, which is the physiological inhibitor of these enzymes [24,25]. NAD+ is also required for cell signalling pathways, serving as precursor for the calcium mobilisers 2″-O-acetyl-ADP-D-ribose (OAADPr), ADP ribose and cyclic ADP ribose. The synthesis of these also liberate nicotinamide [26,27].

**Figure 2:**
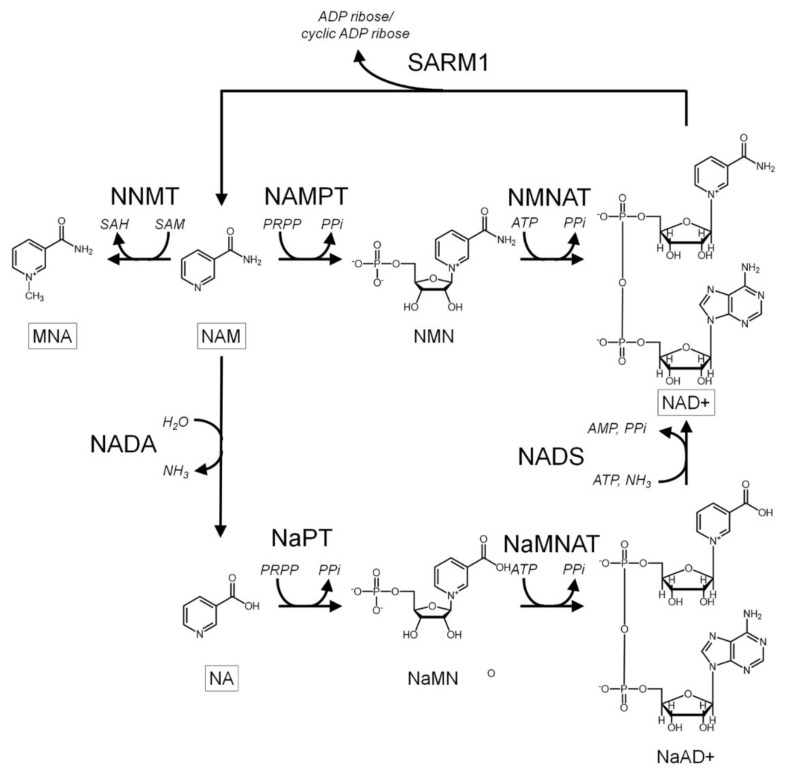
Summary of NAD+ synthesis using nicotinamide as substrate. In vertebrates, NAD+ is synthesised primarily from nicotinamide, obtained either from the diet or the actions of NAD+-consuming enzymes, via the salvage pathway. NAD+ is also cleaved by the NAD+-ase SARM1, releasing nicotinamide plus the signalling molecule (cyclic)ADP-ribose. Levels of nicotinamide are regulated by the *N*-methylation of nicotinamide, producing 1-methylnicotinamide, by nicotinamide *N*-methyltransferase. In invertebrates, NAD+ is synthesised from nicotinic acid, produced via the deamination of nicotinamide by nicotinamidase. The key intermediates in both pathways are boxed. MNA = 1-methylnicotinamide; NAM = nicotinamide; N(a)MN = nicotinamide(ate) mononucleotide; N(a)AD+ = nicotinamide(ate) adenine mononucleotide; NA = nicotinic acid; NNMT = nicotinamide *N*-methyltransferase; N(a)A(M)PT = nicotinamide(ate) phosphoribosyltransferase; N(a)MNAT = nicotinamide(ate) mononucleotide adenylyltransferase; NADA = nicotinamidase; NADS = NAD+ synthase; SAM = *S*-adenosylmethionine; SAH = *S*-adenosylhomocysteine; PPi = pyrophosphate; NH_3_ = glutamine; PPRP = phosphoribosyl diphosphate.

In mammals, NAD+ can be synthesised from tryptophan via the kynurenine pathway; however, this is unable to sustain normal cellular levels of NAD+ [28]. Instead, NAD+ levels in mammals are maintained by synthesis from nicotinamide via the sequential actions of nicotinamide phosphoribosyltransferase (NAMPT) and nicotinamide adenine mononucleotide adenylyltransferase (NMNAT) [29]. Although nicotinamide is available in the diet, the majority (approximately 99%) is derived from the salvage pathway, involving the release of nicotinamide by NAD+-dependent signalling pathways and its reincorporation into NAD+ via NAMPT (Figure 2) [30].

### 2.2. NNMT Function and Regulation of NAD+ Synthesis

NNMT is a 29 kDa cytosolic enzyme which is responsible for the *N*-methylation of nicotinamide into 1-methylnicotinamide (MNA), using *S*-adenosylmethionine (SAM) as methyl donor (Figure 2). The majority of NNMT expression is in the liver, with significant levels found in other tissues such as the brain, kidney, adipose tissue, endothelium, thyroid and pancreas [31,32,33,34,35,36]. Its activity regulates NAD+ synthesis via the reduction in available nicotinamide [37]. NNMT expression is increased in several disease pathologies, including neurodegenerative diseases such as Alzheimer’s [38] and Parkinson’s disease [39,40], hepatic cirrhosis [41], atherosclerosis [42], pulmonary hypertension [43], acute hepatitis [44], fatty liver disease [45], obesity and metabolic syndrome [46,47], peripheral occlusive arterial disease [48] and chronic obstructive pulmonary disease [49]. Several thousand single nucleotide polymorphisms (SNPs) have been reported in the NNMT gene, many of which have been linked to disease and other biochemical outcomes, a full review of which has been published elsewhere [50].

### 2.3. NNMT Promotes the Cancer Phenotype

NNMT expression is significantly increased in many cancers (for a full review see [51]). It is thought that cancer cells arise from transformed stem cells, requiring dedifferentiation to become transformed [52,53]. NNMT has been shown to modulate the epigenetic environment in the differentiation process of stem cells [54,55] and is elevated in cancer stem cells compared to non-tumour cells [55,56,57]. Increased amounts of NNMT alter the methylation status of the genome by reducing available SAM, favouring a pattern of gene expression which promotes the dedifferentiated state and thus drives the evolution of the cell towards a cancer phenotype [56,58,59,60]. Hence, it is likely that NNMT holds a central role in the promotion of the cancer phenotype.

We have investigated the cellular effects of NNMT upon tumour cells using the human neuroblastoma cell line SH-SY5Y. The attraction of using this cell line is that it has no endogenous expression of NNMT, making it an ideal knock-in/knock-out type of in vitro model [37]. We generated a knock-in NNMT-expressing cell line (S.NNMT.LP), which revealed that NNMT is not required for tumour survival; cells continue to grow in the absence of NNMT. Instead, the expression of NNMT provided a survival advantage to the tumour cell by reducing cellular apoptosis and necrosis, decreasing susceptibility to mitotoxins and increasing ATP synthesis [37].

Considering NNMT’s role in regulating NAD+ levels, these cytotrophic effects seem counterintuitive. In our S.NNMT.LP cell line, NNMT expression reduced both cellular NAD+ and NADH content, resulting in an approximately 50% reduction in the NAD+: NADH ratio [37]. It has been suggested that limiting NAD+ availability will counter the beneficial effects of the Warburg effect, likely by restricting the activity of NAD+-dependent enzymes such as NAD kinase which shuttles NAD+ into the PPP [4,29]. Additionally, the activity of DNA repair enzymes such as PARP1 are inhibited by restricted NAD+ availability, which halts tumour progression [29]. However, these reductions in the NAD+:NADH ratio replicate what is observed within cancer cells [61]. Additionally, this reduction was not toxic to the tumour cell, as S.NNMT.LP demonstrated a significant reduction in both apoptosis and necrosis compared to wild-type SH-SY5Y [37].

**Figure 3:**
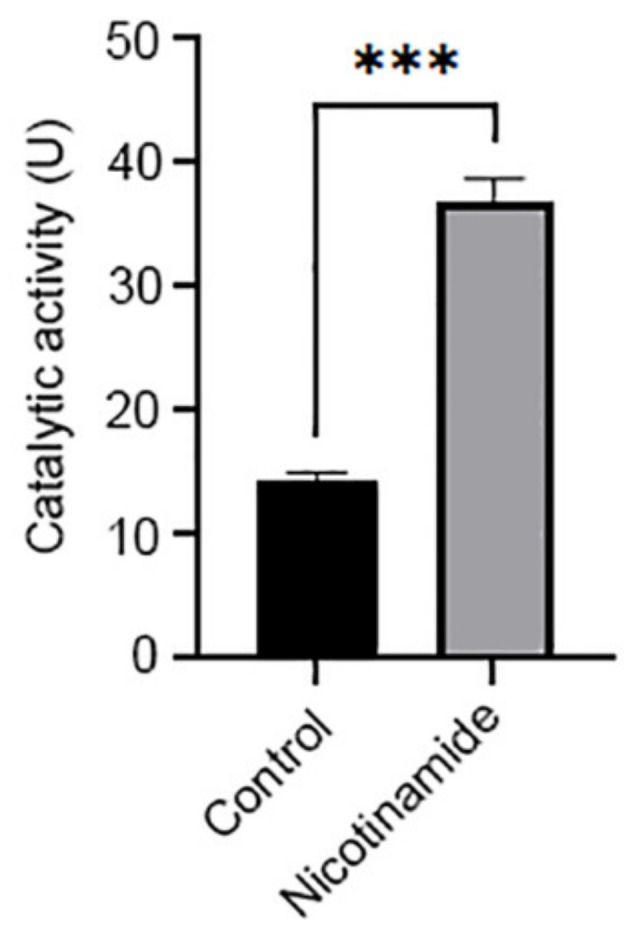
Effect of increased nicotinamide concentration upon NNMT activity in HepG2 hepatocarcinoma cells. Cells were incubated in cell culture media (control) or media supplemented with 100 µM nicotinamide (nicotinamide) for 24 h. Cells were harvested, supernatant prepared and NNMT activity assayed. Results were calculated and expressed as nmol 1-methylnicotinamide produced/hr/mg protein ± S.D. Statistical analysis comprised Student’s *t*-test with Welch correction (*n* = 3). *** = *p* < 0.001.

A recent study by Bockwoldt and colleagues has provided an answer for this paradox. Using mathematical modelling of kinetic constants obtained from published literature for NNMT and NAMPT, they suggested that NNMT and NAMPT co-evolved in vertebrates to maintain NAD+ synthesis whilst maximising the activities of NAD+-dependent pathways essential for the survival of the cell. These pathways produce significant amounts of nicotinamide, which is the physiological inhibitor of NAD+-consuming enzymes [62]. NNMT, with its relative high *k*_m_ for nicotinamide (200 µm) [63], removes the excess nicotinamide generated, thus ensuring that cellular levels of nicotinamide are maintained at low levels, a process which is supported by the induction of NNMT expression by nicotinamide (Figure 3). NAMPT, with its high affinity for nicotinamide as evidenced by its nmolar *k*_m_, can effectively initiate NAD+ synthesis at the low levels of nicotinamide necessary to prevent its inhibitory block of NAD+-consuming enzymes [62]. Thus, by sacrificing a proportion of NADH synthesis, it can be proposed that the tumour cell ensures the activities of NAD+-dependent enzymes can continue at the levels necessary for tumour cell survival and progression by inducing NNMT expression. This cooperation between NNMT and NAMPT is significantly more efficient for the synthesis of NAD+ compared to its synthesis using nicotinic acid via nicotinamidase (NADA), the pathway present in invertebrates such as *Drosophila* as well as yeast and bacteria [62,64].

### 2.4. NNMT Induces Sirtuin Expression and Activity

One of the key outcomes we identified arising from expression of NNMT in S.NNMT.LP cells is an increase in both complex I (CxI) activity and ATP synthesis. This arose due to an increase in the stability of CxI subunits such as NDUFS3 [37]. What is missing is how NNMT mediates these effects. Recent evidence suggests that one such possibility is via sirtuins, DNA histone deacetylases involved in epigenetic regulation which have been implicated in longevity in several in vitro and in vivo models [23,65,66]. They use NAD+ as a substrate, and in common with other NADases their activity is inhibited by nicotinamide. The activation of sirtuins increases mitochondrial function, possibly via the direct deacetylation of subunits of CxI such as NDUFA9 which results in their activation [67]. The co-regulation of NNMT and sirtuin expressions and activities were first linked by Estep and colleagues who demonstrated that calorie restriction in mice increases the expression of both NNMT and sirtuin-1 [68]. We demonstrated that the expression of NNMT in S.NNMT.LP cells increased the amount of sirtuin 1 and 3 proteins which we showed, using siRNA silencing of sirtuin-3 expression, mediated the increased CxI activity and cellular ATP synthesis we observed. Furthermore, the increased amount of sirtuin protein was accompanied by the activation of sirtuin-3 activity as evidenced by the reduction in the acetylation of the SH-SY5Y proteome, an effect most likely mediated via the removal of nicotinamide inhibition by NNMT [69]. Subsequent studies have shown that MNA binds directly to sirtuins, via an as-yet unidentified binding site, preventing its degradation and thus maintaining cellular sirtuin protein levels [70,71,72,73]. In support of this, we demonstrated that incubation of SH-SY5Y cells with MNA increased both CxI activity and ATP synthesis, and increased NDUFS3 stability, an effect likely due to the interaction of MNA with sirtuins [37].

### 2.5. NNMT Increases Mitochondrial Function

Mitochondrial activity is still essential for the generation of NAD(P)H and FAD to support the Warburg effect [12,13]. Sirtuins regulate the expression of several downstream pro-mitochondrial genes such as uncoupling protein-2 (UCP2), peroxisome proliferator-activated receptor-γ (PPAR-γ) and PPAR-γ coactivator 1-α (PGC1α) [74,75]. UCP2 is a mitochondrial uncoupling protein, resident in the inner mitochondrial membrane, which dissipates the proton motive force by allowing protons to flow down their concentration gradient back into the mitochondrial matrix. The consequence of this uncoupling is to reduce oxidative stress-mediated generation of reactive oxygen species (ROS) [76,77]. PPAR-γ is a nuclear receptor which activates the transcription growth factor-β signalling [78] and the mammalian target of rapamycin (mTOR) [79] pathways and interacts with PGC1α [80]. PGC1α is a transcriptional coactivator which regulates the expression of genes involved in energy metabolism and is the master regulator of mitochondrial biogenesis [81,82]. Hence, NNMT may induce pro-mitochondrial changes in gene expression and subsequent mitochondrial activity which support the Warburg effect. This is evidenced by the 2-fold increase in ATP synthesis we observed in S.NNMT.LP cells, which arose from an increase in mitochondrial activity as evidenced by a 300% increase in CxI activity and a 60% increase in oxygen consumption [37]. Whilst these increases seem somewhat large and out of step with the concept of reduced reliance upon mitochondrial generation of ATP, they are relative to activities observed in wild-type SH-SY5Y cells, which are cancer cells and thus rely predominantly upon cytosolic ATP production. Hence, the absolute increases in these mitochondrial measures are relatively small and indicate a maintenance of mitochondrial activity rather than a wholesale activation of mitochondrial function. This fits well with the hypothesis that mitochondrial function is primarily supporting NAD(P)H and FAD recycling to support the increase in the activities of the PPP and lipid biosynthetic pathways, rather than to increase ATP synthesis. The enhanced mitochondrial function we have reported correlates with other studies which have shown activation of mitochondrial activity in cancer [13].

A further potential effect of increased NNMT expression is a reduction in oxidative stress arising from the rapid proliferation of the tumour biomass. Although ROS generation is essential for the progression of the tumour phenotype [83,84,85,86] and the promotion of tumour growth [87,88], the tumour cell also needs to protect itself against the increased production of ROS to prevent the activation of apoptotic pathways and thus tumour cell death [89,90,91]. Hence, the tumour must navigate a fine balance between the beneficial and deleterious effects of enhanced ROS production and thus must keep ROS levels within a narrow window to ensure survival and proliferation [92,93]. Tumour mitochondria are a major source of such ROS [88,92,94,95], and ROS protective mechanisms such as enhanced glutathione levels have been shown to be increased in many tumours [92,93,96]. The much higher increase in CxI activity compared to ATP generation and oxygen consumption in our S.NNMT.LP cell line suggests a degree of uncoupling of the mitochondrial respiratory chain, as evidenced by increased expression of UCP2 protein in S.NNMT.LP cells. The increase in the expression of UCP2 correlated with decreased markers of oxidative stress such as superoxide and isoprostane F2α, plus an increase in the GSH:GSSG ratio [97]. The expression of UCP2 is increased in cancer and in cancer cachexia in response to increasing oxidative stress, the consequence of which is an increase in their apoptotic threshold [77,98,99,100,101]. Hence, the expression of NNMT has a 2-fold beneficial effect on the mitochondria, the first to maintain their function to support the Warburg effect, and the second to assist in maintaining ROS generation within a pro-oncogenic range.

### 2.6. NNMT Activates the Akt Signalling Pathway

A key feature of cancer metastasis is the induction of the Akt pathway [102]. Akt, also known as protein kinase B, is a component of the mTOR pathway and is central to the regulation of a number of fundamental cell signalling pathways involved in survival, proliferation, drug resistance and differentiation [103]. Induction and activation of the Akt signalling pathway, along with the presence of gain of function mutations, are observed in a wide number of cancers including thyroid, breast, colon and pancreatic cancers [104] and as such aberrant Akt signalling is highly oncogenic [105,106]. Akt signalling is induced by NNMT [107,108], which is mediated by the induction and cleavage of ephrin B2 [108] and subsequent binding to the ephrin B receptor [109].

The consequences for NNMT-mediated induction of Akt activity are significant for the survival and proliferation of the tumour cell. In clear renal cell carcinoma (cRCC) cells, increased Akt signalling induces the expression of matrix metalloproteinase-2, the consequence of which is the remodelling of the extracellular matrix and the subsequent promotion of tumour invasion during metastasis [107]. Activation of the Akt signalling pathways reduces cellular apoptosis by inhibiting p53, reducing the expression of the pro-apoptotic protein Bim and increasing the translation of anti-apoptotic genes such as glycogen synthase kinase-3 [110]. In line with these activities, reduced apoptosis is observed in both S.NNMT.LP cells [37] and in human breast cancer cells [111].

Akt also has several other mitochondrially-targeted effects in cancer. Akt induces hexokinase expression, necessary to phosphorylate the increased levels of glucose entering the cell via the glucose transporter due to the Warburg effect. Additionally, mitochondrial Akt binds to mitochondrial porin, which redirects mitochondrial ATP to support the increase in hexokinase-mediated phosphorylation of glucose [112]. The mitochondrial pool of Akt also promotes tumour development by phosphorylating the mitochondrial calcium uniporter, resulting in increased levels of mitochondrial calcium [113].

The activation of the Akt pathway has been shown to be a central regulator of drug resistance in cancer, arising from interactions with pro-survival pathways such as the MAPK, Notch and Wnt/β-catenin signalling pathways [114]. Inhibition of the Akt pathway has been shown to overcome resistance to cisplatin in a variety of cancer types [115,116,117,118], hence targeting the Akt signalling pathway is an attractive target for current cancer therapy studies [114]. To date, a small number of studies have investigated the effect of NNMT upon drug resistance in cancer. We have shown that NNMT protects against mitochondrial toxins in S.NNMT.LP cells [37,119,120,121]. NNMT induces resistance of colorectal cancer cells to 5-fluorouracil (5-FU) [122] and has been implicated in the resistance of cervical squamous cell carcinoma to treatment [123]. Increased expression of NNMT in non-small-cell lung cancer cells results in resistance to EGFR-tyrosine kinase inhibitors [124]. To date, no study has linked these effects to increased phosphorylation and thus activation of Akt. Although pathways such as the ASK1-p38-MAPK pathway have been implicated in NNMT-mediated resistance of colorectal cancer cells to 5-FU [125], given the strong cytoprotective effects of Akt signalling, along with the well-documented regulation of Akt phosphorylation demonstrated by us and others in a wide variety of cancers, it is likely that the NNMT–Akt axis is heavily involved in acquired drug resistance.

## 3. NNMT Promotes the Epithelial-to-Mesenchymal Transition

A key role for NNMT in the cancer cell is the promotion of the epithelial-to-mesenchymal transition (EMT), which involves the conversion of epithelial cells into mesenchymal cells characterised by an increase in stem cell phenotype and behaviour [126]. This process is essential for the development of increased invasiveness and thus promotion of metastasis [127]. This in turn mediated the activation of matrix-degrading enzymes such as matrix metalloproteases [128]. An additional effect of the EMT is to reduce apoptosis [129]. As discussed earlier in this review, increased NNMT expression in cancer cells replicates all these effects, demonstrating the central importance of NNMT expression to the EMT. This was elegantly demonstrated recently by Eckert and colleagues who showed that NNMT was central in driving the transition of non-neoplastic fibroblasts into cancer-associated fibroblasts, a process which they successfully prevented therapeutically in an in vivo model of ovarian cancer [59]. For a more complete review of this topic, please see [130].

## 4. Linking NNMT with Its Cellular Functions

### 4.1. Epigenetic Regulation

One key question is how to link increased NNMT expression with its functions within the cell. One such mechanism is via epigenetic regulation arising from reduction in intracellular SAM concentration. SAM is the methyl donor for all methylation reactions and is produced by the adenylation of methionine by methionine adenylyltransferase [29]. Methyltransferases are found in diverse pathways such as Phase II metabolism [131,132], the regulation of protein activity [133,134] and the regulation of gene expression [135,136]. DNA methylation is a key process in regulating gene expression, in which methylation of CpG islands prevents expression of genes [135]. Due to the sole requirement for SAM as a cofactor in methylation reactions, the regulation of DNA expression is therefore heavily influenced by SAM availability.

There is a growing body of evidence which suggests that NNMT regulates the expression of pro-oncogenic genes in cancer via the regulation of intracellular SAM concentrations. By reducing available SAM levels, NNMT reduces DNA CpG island methylation, the consequence of which is enhanced gene expression. For example, increased NNMT expression in pancreatic cancer creates a “metabolic sink” whereby intracellular SAM levels are reduced due to NNMT overactivity, the consequence of which is the upregulation of the expression of several cancer-related genes such as *SNAI2*, *ADAMTS6*, *TGFB2*, *LAMB3* and *CNTN1* [137]. NNMT expression replicates many of the effects of these genes, such as promoting the EMT, extracellular matrix remodelling and activation of cell signalling pathways [59], hence NNMT-mediated changes in epigenetic regulation are likely responsible for some of its pro-tumour effects.

### 4.2. Regulation of NAD+-Dependent Pathways

NNMT also influences gene expression indirectly via the regulation of intracellular NAD+ and nicotinamide levels. Pathways which use NAD+ as a substrate are also inhibited by nicotinamide, the by-product of NAD+ cleavage. As described earlier in this review, NNMT co-evolved with NAMPT to maximise the activity of NAD+-dependent enzymes despite increasing levels of nicotinamide arising from their activity [62]. Many of these pathways are pro-survival and their activities are dysregulated in many cancers. One such example is sirtuins. Another example is PARP-1. PARP-1 is an NAD+-dependent DNA repair enzyme whose primary activity is the repair of both single-strand and double-strand breaks [138]. When DNA damage is too high, PARP-1 initiates cell death rather than DNA repair [139], the fate of which is determined by the availability of the intracellular NAD+ pool [140]. Patients with bladder cancer undergo nicotinamide infusion prior to radiotherapy to inhibit DNA repair by PARP-1. Increased NNMT expression reduces the efficacy of radiotherapy by removing the inhibitory block of nicotinamide upon PARP-1 [141,142,143]. PARP-1 also positively regulates the expression of many pro-oncogenic genes via the PARylation of DNA histones [144]. Inhibition of PARP-1 is an emerging therapeutic target in cancer therapy [138], thus its activation by the reduction in cellular nicotinamide levels by NNMT may contribute to the pro-oncogenic effect of PARP-1.

### 4.3. Synthesis of 1-Methylnicotinamide

One possibly mediator of the effects of NNMT is its product of nicotinamide *N*-methylation, MNA. Long considered merely an excretion product, a considerable body of evidence suggests that it has pharmacological activity in conditions ranging from inflammation and hepatotoxicity through to thrombosis and chronic obstructive pulmonary disease [41,145]. The first evidence for an important pharmacological activity of MNA was reported by Chlopicki and colleagues [145], in which they demonstrated a dose-dependent thrombolytic effect in Wistar rats at doses ranging from 30–100 mg/kg. This thrombolytic response was independent of changes in arterial blood pressure; instead, the effects of MNA were mediated via an increase in cyclooxygenase-2 (COX-2)-mediated release of prostacyclin (PGI_2_). MNA reduced apoptosis of SH-SY5Y human neuroblastoma cells in a dose-dependent manner [37], in addition to replicating many of the effects of NNMT expression upon tumour-promoting biochemical changes such as increased ATP synthesis and CxI activity, plus changes in cell morphology [37,108], all cognisant with changes observed during metastasis in cancers such as colorectal carcinoma [146,147,148] and breast cancer [149]. Elevated MNA levels correlate with lower survival rate and shorter life expectancy of patients with cervical cancer and thus most likely contributes to the disease progression [150]. Increased MNA synthesis in colorectal cancer xenographs in vivo increases their resistance to 5-FU via the inhibition of apoptosis, which is mediated by the attenuation of the ASK1-p38 MAPK pathway [125]. MNA also binds directly to sirtuin-1 and stabilises it, thus increasing its activity. One consequence of this stabilisation is the enhancement of chemoresistance of breast cancer cells to the anti-tumour agents adriamycin and paclitaxel [70]. Currently, the location of this allosteric site for MNA is unknown.

In contrast, a recent study showed that the role of MNA in promoting tumour development may not be as clear cut as first thought. Despite enhancing tumour vasculature formation in 4T1 murine mammary gland tumours, MNA inhibited metastatic development in the lungs of mice ectopically inoculated with 4T1 cells. Furthermore, when co-administered with the cytostatic anti-tumour agent cyclophosphamide, lung metastases were reduced by 80% [151]. It is also possible that MNA may exert its anti-metastatic effect via increased release of PGI2 [145], a molecular which has been shown to prevent metastasis in a number of in vitro and in vivo models [152,153,154]. The reasons for such discrepancies between studies is unclear but may reflect differences in tumour types investigated and analysis methodologies employed. It does raise the possibility, however, that MNA may have a mixed mode effect upon tumour progression and survival. For example, it is possible that the pro-tumour effects of MNA arises from its binding and consequent stabilisation and activation of sirtuins [70,71,72,73], the consequences of which for the tumour cell include pro-oncogenic changes in epigenetic regulation and protein acetylation [22,23] along with enhancement of chemoresistance [70]. Anti-tumour effects of MNA may at the same time arise from its inhibition of NNMT activity [1,31,63,155] and its subsequent effects upon nicotinamide levels, NAD+ synthesis and cellular SAM availability, the consequences of which include decreased activity of NAD+-dependent pro-oncogenic enzymes such as PARP-1 [141,142,143,144] and reversal of pro-oncogenic changes in epigenetic regulation [56,58,59,60], as well as increased production of PGI_2_. What is clear is that further research is required to fully elucidate the role of MNA in tumour survival and progression.

## 5. Mechanisms Underlying Increased NNMT Activity in Cancer Cells

### 5.1. Transcription Factor Binding Sites in the NNMT Gene

**Figure 4:**
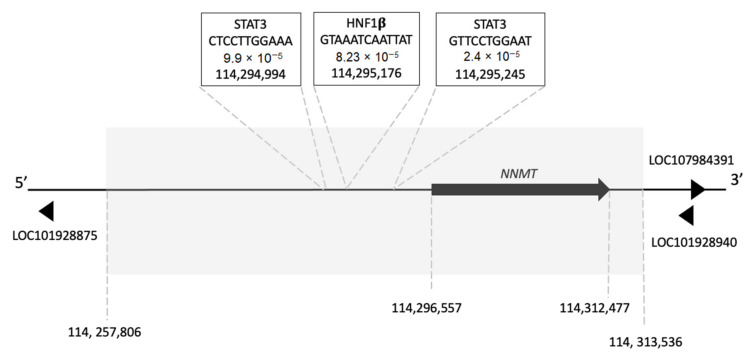
Schematic representation of the *NNMT* genomic neighbourhood showing the position of putative STAT3 and HNF-1 transcription binding sites (TFBS) and three lncRNAs. Sites were predicted in FIMO with *p*-values for each predicted TFBS given below the retrieved motif. Coordinates of all features taken from GRCh38.p13 (accession NC_000011.10). lncRNAs are indicated with “LOC” numbers. Shaded area = *NNMT* gene regions. Arrows are not drawn to scale.

Despite the widely recognised importance of NNMT overexpression in cancer, few studies have investigated how NNMT expression is regulated. It is known that the coding domain sequence produces two transcripts, NNMT-201 and NNMT-203, and these encode the same translated protein of 264 amino acids. However, NNMT-201 and NNMT-203 differ in their transcription initiation sites (TIS). In addition to these two coding transcripts, the latest version of the human genome (GRCh38.p13. refseq accession NC_000011.10) indicates that there are an additional three transcript variants of *NNMT* which appear to be shorter in length than both NNMT-201 and NNMT-203. Their role is unclear, but they may play a role in the regulation of mRNA processing [50]. Scrutiny of the genomic neighbourhood surrounding *NNMT* also reveals three, long non-coding RNAs (lncRNA) (LOC101928875, LOC101928940 and LOC107984391) that are highlighted here for the first time. All three of these lncRNAs are within the *NNMT* genomic neighbourhood, although LOC101928875 is approximately 97 kb upstream of *NNMT* and is located within another gene region. LOC101928940 and LOC107984391 are approximately 47 kb and 59 kb downstream of *NNMT*, respectively (Figure 4). Whether these three lncRNAs play any role in the regulation of *NNMT* expression is so far unknown and is yet to be investigated.

Within the *NNMT* promoter region, which has been proposed to extend 2 kb upstream of the *NNMT* coding domain sequence [50], several putative transcription factor binding sites (TFBS) have been identified [36,156,157,158]; however, only the presence of STAT3 and HNF-1β regulatory sites have been experimentally demonstrated [36,156,159]. We have subsequently performed a more in-depth bioinformatic analysis of the *NNMT* gene to identify the location of the two known TFBSs, STAT3 and HNF-1β, both within its 5′-flanking region and introns. Using position weighted matrices of STAT3 (MA0114.2) and HNF-1β (MA0153.2) from JASPAR [160], the *NNMT* promoter region and introns were scrutinised for putative TFBS using FIMO [161] implemented in the MEME Suite [162]. In agreement with our previous work [50] we identified, with high probability, two putative STAT3 recognition sites (GTTCCTGGAAT and CTCCTTGGAAA) at positions 114,295,245 and 114,294,994 plus a putative HNF-1β recognition site (GTAAATCAATTAT) at position 114,295,176. We also identified further putative STAT3 and HNF-1β TFBS in this region but disregarded them due to either low probability scores or because they were found some distance away from the gene itself.

The presence of several STAT3, and a single HNF-1β TFBS in the *NNMT* promoter region suggest a mechanism for the observed induction of NNMT expression in cancer. STAT3 is constitutively activated in many cancers, promoting cancer progression, proliferation, metastasis [163,164] and drug resistance via the regulation of signalling pathways including Akt [165,166,167]. Overexpression of HNF-1β has been implicated in tumour progression in cancers such as clear cell carcinoma of the ovary, liver, pancreas, kidney, endometrium, and prostate [168,169,170,171,172]. HNF-1β leads to the development of chemoresistance to carboplatin in clear cell ovarian cancer via the upregulation of glutathione synthesis [173] and has also been suggested to promote the Warburg effect and ROS reduction [174]. Interestingly, our analysis also identified several putative STAT3 and HNF-1β TFBSs throughout introns 2 and 4 (Table 1); however, whether these sites are functional is yet to be determined.

### 5.2. NNMT Single-Nucleotide Polymorphisms and Cancer

Over 12,000 SNPs are currently known for the *NNMT* gene, many of which are associated with either disease or other physiological conditions such as increased athletic performance (for a full, in-depth review of this topic, please see [50]). In total, 21 different cancers have a somatic SNP in the *NNMT* gene; however, only 102 SNPs are represented in a combined 119 instances of cancer, thus it is impossible to determine whether SNPs are causative or coincidental. Additionally, functional confirmation of the effects of these SNPs upon protein activity, protein expression or mRNA stability is lacking [50].

### 5.3. Post-Translational Modifications

Post-translational modification of proteins regulates diverse functions such as enzyme activity, protein folding, cellular trafficking, and protein-protein interactions [175,176]. However, very little research into the post-translational modification of NNMT in both healthy and cancerous tissues, and in non-cancer diseases, has been undertaken. The first post-translational modification to be functionally confirmed for NNMT was phosphorylation in gastric tumours by casein kinase 2 (CK2). The consequence of this phosphorylation for NNMT was not determined. Subsequent eukaryotic linear motif searching of the NNMT sequence revealed several further potential modification sites, including phosphorylation consensus sequences for glycogen synthase kinase 3, protein kinase A and protein-directed kinase (a component of the MAPK pathway) along with predicted sites for glycosaminoglycation and sumoylation [177]. Further analysis, using the PhosphoSitePlus v.6.5.9.3 webtool [178], predicts several further potential post-translational modification sites, including acetylation, ubiquitinylation, succinylation and methylation (Table 2). However, the experimental confirmation of these post-translational modification sites has yet to be undertaken.

CK2 is implicated in the proliferation a wide variety of tumours, including gastric tumours [180], and to promote chemoresistance to anti-tumour agents such as paclitaxel [181]. CK2 inhibition by CX-4945 mitigates the Warburg effect [180], an effect possibly mediated at least in part via reduced phosphorylation of NNMT. MAPK [182], PKA [183] and GSK3 [184] signal transduction pathways are all implicated in tumour progression and resistance. Thus, functional confirmation of the presence of these regulatory sites in the NNMT protein sequence will provide further insight into the promotion of the cancer phenotype by NNMT.

Citrulline is a non-coding amino acid which is produced via the deamidation of protein arginine residues by protein arginine deiminases (PAD) [185], resulting in the production of a positively-charged residue [186,187]. Nemmara and colleagues revealed the presence of three citrullination sites, R18, R132 and R181, in the NNMT protein sequence. Of the three sites, citrullination of R132 resulted in reduced SAM binding via the disruption of the conformation of loop 7 of the NNMT structure, the consequence of which was the inactivation of NNMT activity. The citrullination of the remaining two sites had no effect upon NNMT activity [179]. Abnormal PAD activity is observed in many cancers such as triple-negative breast cancer [188], hence this may mediate the effects of NNMT observed in many cancers.

## 6. NNMT and Cancer—Cause or Consequence?

Accumulating evidence is pointing to a central role for NNMT in the correct functioning of the cell, many of which are hijacked by the cancer cell to promote growth and survival. The predicted co-evolution of a high *k*_m_ NNMT alongside a low *k*_m_ NAMPT in vertebrates [62,64] raises the question—has the evolution of NNMT driven the evolution of the cancer phenotype? Cancer cells appear to have taken advantage of the NNMT/NAMPT pathway to promote the neoplastic phenotype by increasing the activity of NAD+-dependent processes such as sirtuins and PARP-1. Mitochondrial function is also stimulated, most likely to provide the NAD+ and FAD required to maintain glycolysis and the Krebs cycle as part of the Warburg effect. Finally, Akt signalling is also activated, the consequence of which is the stimulation of anti-apoptotic processes and the induction drug resistance, an effect also induced by NNMT-mediated stabilisation of sirtuin-1 via MNA.

**Figure 5:**
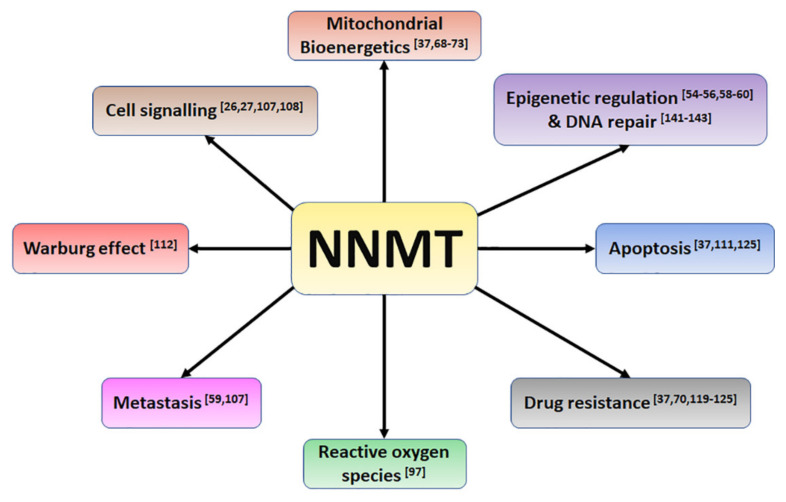
Summary of the pro-oncogenic cellular effects of NNMT expression. Numbers represent citations in the text.

A chain of events can therefore be envisioned which can describe the metabolic changes occurring because of increased NNMT expression in the tumour cell. Upon neoplastic transformation, there is as shift in ATP generation towards glycolysis [1]. Despite a reduction in the reliance upon the mitochondria for ATP production, there is still a need to replenish the large amounts of NAD+ and FAD being consumed by glycolysis and the PPP, which are obtained from the mitochondria [10,11]. Increased NNMT expression contributes towards meeting this need by increasing mitochondrial function [37]. As part of the neoplastic transformation, the increased activities of NAD+-dependent pro-oncogenic enzymes such as PARP1 and sirtuins generate significant amounts of nicotinamide, the physiological inhibitor of NAD+-consuming enzymes [62], thus the consequence of this is the inhibition of their respective activities. NNMT prevents this by *N*-methylating nicotinamide, thus removing this inhibitory block, whilst ensuring NAD+ synthesis occurs at optimal levels via the NMNT/NAMPT axis [62,64]. Finally, NNMT expression also increases Akt phosphorylation [107,108], thus enhancing pro-oncogenic signalling within the cell, resulting in effects such as the promotion of metastasis [107], inhibition of apoptosis [37,111], promotion of drug resistance [114,119,120,121] and supporting glycolysis by increasing hexokinase expression [112]. Hence, NNMT sits at the centre of a web of cellular processes, each of which contribute to the promotion of the neoplastic phenotype by supporting the metabolic shift of the cell towards glycolysis whilst maintaining NAD+ synthesis and signalling as well as inducing pro-oncogenic processes (Figure 5).

One way to address this question is to observe whether cancer affects those species which do not express NNMT but use NADA instead, for example *Drosophila.* NADA hydrolyses nicotinamide, producing nicotinic acid which then enters the NAD+ synthetic pathway via nicotinic acid adenylyltransferase (NaMNAT) [189,190] (Figure 2). What is key about this pathway is that it does not use SAM, thus one of the major routes by which NNMT influences the cancer phenotype—epigenetic regulation via reduced DNA methylation—is absent. *Drosophila* cancer models are routinely used to reproduce aspects of human cancers such as metastasis, cachexia and drug resistance [191,192]. The presence of the Hippo signalling pathway in *Drosophila*, and its dysregulation in many human carcinomas, makes them a simple yet effective model of human cancers [193]. Most importantly, *Drosophila* develop naturally occurring tumours [194]. Taken with the fact that NNMT expression is not required for a basic cancer phenotype [37], it is likely that NNMT plays no role in the initiation of tumorigenesis but instead is recruited as part of an evolution of the tumour phenotype to provide a survival advantage over surrounding non-cancerous cells. This is evidenced by (1) pro-oncogenic changes in epigenetic regulation [59], promotion of the activities of pro-oncogenic NAD+-dependent pathways such as sirtuins and PARP-1 [69,141,142,143], maintenance of mitochondrial function and support of the Warburg effect [37,97,195], activation of pro-oncogenic signalling pathways such as Akt [107,108] and protection against anti-cancer drugs [37,119,120,121,122,123,124,125]. We have shown that SH-SY5Y cells expressing NNMT demonstrate reduced cell death in response to toxins compared to wild-type SH-SY5Y which have no endogenous NNMT expression [37,119], and as discussed elsewhere in this review, numerous other studies have demonstrated increased drug resistance in response to increased NNMT expression.

## 7. Therapeutic Targeting of NNMT

**Figure 6:**
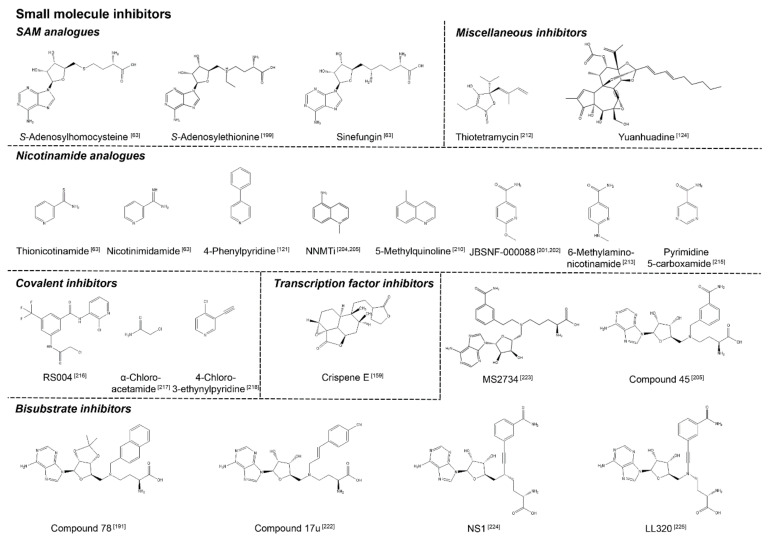
Structures of inhibitors of NNMT activity and expression. Numbers in italics are citations reporting NNMT inhibitory activity or downregulation of expression.

With such a central role in the regulation of cellular processes underlying cancer progression, it is no surprise that NNMT is now a focus of intense cancer drug studies [130]. The ability to target a specific enzyme to reverse many pro-oncogenic pathways in a single hit is very attractive. Such approaches which target for example transcription factors have the potential for significant off-target effects due to their ubiquitous nature in many tissues and their involvement in essential cellular functions for non-tumour cells [196,197]. The drawback to NNMT-targeted therapy is that it is unlikely to act as a stand-alone drug. Evidence for this lies with those tumours in which NNMT is not a feature, for example human neuroblastomas [37]. This seeming weakness is probably its greatest strength, as it suggests that off-target effects for NNMT-based therapies will be minimal. It is therefore likely that the best use for NNMT-based cancer therapies is as an adjunct to anti-tumour drugs, acting to prevent tumour metastasis and burden and to reduce the survival advantage afforded to tumour cells by increased NNMT expression. Currently, two general approaches are being investigated, small-molecule inhibitors of NNMT activity and small-molecule inhibitors of NNMT expression (Figure 6).

### 7.1. Small-Molecule Inhibitors of NNMT Activity

Many research groups, including our own, are now actively developing inhibitors of NNMT as anti-cancer therapies (for a comprehensive review of the current state of NNMT inhibitor development, see [198]). The first description of an NNMT inhibitor which was not *S*-adenosylhomocysteine (SAH) or MNA identified *S*-adenosylethionine as a potent inhibitor, although this was significantly less effective than SAH [199]. In recent years, rapid progress in inhibitor design has been made in a relatively short amount of time due to our increased understanding of the interactions between nicotinamide, SAM and the NNMT active site [30,198]. The key to unlocking this understanding was the first publication of the crystal structure of NNMT co-crystalised with nicotinamide and SAH by Peng and colleagues [200]. The majority of the first small-molecule inhibitors developed were focussed upon targeting NNMT in obesity and alcohol-related fatty liver disease [201,202,203,204,205]. Many of these inhibitors have yet to be tested in in vitro cancer models systems, and questions remain about their selectivity towards NNMT in relation to other methyltransferases as well as their cell permeability. This is particularly important for NNMT’s family members phenylethanolamine *N*-methyltransferase (PNMT), responsible for the conversion of noradrenaline to adrenaline [206], and indolethylamine *N*-methyltransferase (INMT), which is involved in tryptamine and serotonin metabolism [207].

### 7.2. Nicotinamide Analogues

The active site of NNMT is relatively large, containing three binding pockets, one for nicotinamide, one for the adenosine of SAM and one for the amino acid portion of SAM [198,208]. This at first glance would appear to be beneficial, as it provides a large canvas for designing inhibitors which bind to these pockets. However, the drawback is the ubiquitous use of SAM as the methyl donor in methylation reactions and their use in almost all areas of cell function and survival. Inhibitors such as SAH and sinefungin, a close structural analogue of SAM, are non-selective and inhibit all methyltransferases, including NNMT [63].

Initial studies of NNMT substrate specificity revealed a wide substrate profile [209], which for a long period of time was the basis for labelling NNMT merely as an enzyme of Phase II metabolism. The poor rates of *N*-methylation of many of these substrates would indicate that they are in fact competitive inhibitors of NNMT. In a recent study, we performed a wide-ranging screen of potential NNMT substrates revealed that the majority were poor substrates. The catalytic efficiencies ranged from 59% to 4% for the close structural analogues thionicotinamide and nicotinimidamide, respectively [63]. The *k*_cat_ for the most poorly performing, nicotinimidamide, was 0.0135 s^−1^, which equates to a turnover time of approximately 71 s to *N*-methylate one molecule, suggesting activity more closely related to that of an inhibitor. A further poor substrate, 4-phenylpyridine (4-PP) [63], demonstrated substrate inhibition kinetics arising from its 180° rotational symmetry of binding in the NNMT active site. Furthermore, the *k*_cat_ was 0.00057 s^−1^, a turnover time of 1754 s or approximately 30 min per molecule [121]. What these studies show is that molecules which bind to the active site can provide significant inhibition of activity. However, the high concentrations of inhibitor required to elicit inhibition, due to the high *k*_i_ of substrate-inhibitors such as 4-PP (4 mM) [121] and high *k*_m_ for compounds such as nicotinimidamide compared to nicotinamide (1.3 mM vs. 0.2 mM) [63], make these small-molecule analogues unsuitable.

Others have had more success. The first successful inhibitor to be reported was NNMTi, which was shown to reverse high-fat diet-induced obesity in mice [204,205]. NNMTi has an inhibitory concentration (IC_50_) of 1.2 μM. In human cancer-associated fibroblasts, NNMTi increased histone methylation and tubulin acetylation, and in an in vivo model of ovarian cancer metastasis it reduced tumour burden. It is important to note that NNMTi did not affect the viability of CAFs or ovarian cancer cells [59], thus supporting the hypothesis stated earlier in this review that NNMT-targeted drugs may prevent tumour metastasis and thus burden but not induce tumour cell death. Crucially, all these effects were specific to NNMT-expressing cells, suggesting that off-target effects with such an approach would be minimal [59].

5-Methylquinoline is a small-molecule inhibitor of NNMT which selectively inhibits HeLa cell proliferation without having any effect upon HEK-293 proliferation [210]. This selectivity towards the inhibition of tumour rather than stem cell proliferation suggests that NNMT inhibitors are likely to be selective for tumour cells, thus reducing the possibility of deleterious off-target effects for non-tumour tissues. Yuanhuadine (YD) is a naturally derived anti-tumour agent used for the treatment of cancers of the lung [211]. YD binds to the nicotinamide binding site of NNMT, resulting in inhibition with an IC_50_ of 400 nM. YD also reversed NNMT-mediated resistance of non-small cell lung cancer cells to 5-FU [124]. The anti-bacterial compound thiotetramycin and its derivatives demonstrated mild inhibitory effects against NNMT [212]. JBSNF-000088 (a substrate inhibitor) [201,202], 6-methylaminonicotinamide [213] and methylated quinolinium analogues such as 5-amino-1-methylquinoline [34,214], are nicotinamide analogues which all have IC_50_ values in the single μM range. Recent studies have described inhibitors with IC_50_ values in the low nM range [202], such as pyrimidine 5-carboxamide which has an IC_50_ of 74 nM [215]. However, to date, none of these have been tested in any in vitro or in vivo cancer models.

### 7.3. Covalent Inhibitors

A small number of studies have developed covalent inhibitors of NNMT, which bind to the active site and prevent access for nicotinamide. Such inhibitors have been developed primarily as pharmacological tools for investigating methyltransferase enzymes, rather than as therapeutic treatments for disease. The first to be developed, RS004, is based upon the structure of SAH, and covalently links to Cys165 in the NNMT active site. This residue is unique to NNMT, thus providing selectivity towards NNMT. RS004 demonstrated NNMT inhibitory efficacy with an IC_50_ of 1 μM [216]. Lee and colleagues produced α-chloroacetamide as inhibitors with low μM activities; however, cellular efficacy and specificity towards NNMT were low [217]. 4-chloro-3-ethynylpyridine is a suicide inhibitor of NNMT, requiring prior *N*-methylation by NNMT before covalently binding to Cys159. The IC_50_ of this molecule was 36 μM [218]. Covalent inhibitors represent approximately 30% of drugs currently in clinical use and have many benefits, such as high potency, less frequent dosage, wide therapeutic windows, and reduced development of drug resistance. Such inhibitors are rapidly becoming more abundant in cancer drug design [218]. However, they do come with increased risk of toxicity and consequences from off-target effects [219,220]. As yet, no covalent NNMT inhibitor has been screened for specificity for NNMT over its family members PNMT and INMT, instead relying on the unique cysteine target residue in the NNMT active site for their selectivity. Furthermore, none have been tested in an in vivo model, therefore the potential for their toxicity is unknown.

### 7.4. Bisubstrate Inhibitors

Recent efforts have focussed upon the design and synthesis of bisubstrate inhibitors. Such compounds comprise, within a single molecule, moieties designed to bind to both the nicotinamide and SAM binding sites. In this manner, it is proposed that substrate inhibitors increase the inhibitory efficacy and the enzyme selectivity over that possible with small molecule, nicotinamide analogue-type inhibitors. Using this approach, inhibitors with low EC_50_ values have been successfully developed, some of which demonstrate efficacy in in vitro cancer models and increased specificity towards NNMT.

Taking advantage of our novel UHP-HILIC-MS-based NNMT activity assay [63], we designed and synthesised the first bisubstrate inhibitors which mimicked the NNMT methylation reaction transition state with µM efficacy. The lead compound, the trivalent molecule (S)-2-amino-4-((((2R,3S,4R,5R)-5-(6-amino-9H-purin-9-yl)-3,4-dihydroxytetrahydrofuran-2-yl)methyl)(3-carbamoyl-benzyl)-amino)butanoic acid (compound 45), binds to the nicotinamide, adenosine and amino acid binding pockets of the active site, and is a potent inhibitor of NNMT with an IC_50_ (29 µM) on par with that of MNA (25 µM) and sinefungin (17 µM) [205]. Further optimisation of the nicotinamide moiety and the linker connecting the amino acid moiety of compound 45 resulted in a compound, (S)-2-Amino-4-((((2R,3S,4R,5R)-5-(6-amino-9H-purin-9-yl)-3,4-dihydroxytetrahydrofuran-2-yl)methyl)(napthalen-2-ylmethyl)amino)butanoic acid (compound 78) with an IC_50_ of 1.7 µM. Furthermore, compound 78 inhibited the proliferation of HSC-2 human oral cancer cells [221]. At the time of writing, the specificity of these inhibitors for NNMT over other methyltransferases has yet to be published. Our most recent inhibitor, (S)-2-amino-4((((2R,3S,4R,5R)-5-(6-amino-9H-purin-9-yl)-3,4-dihydroxytetrahydrofuran-2-yl)methyl)((E)-3-(4-cyanophenyl)allyl)-amino)butanoic acid (compound 17u), replaces the nicotinamide mimic common in current bisubstrate inhibitors with an electron-deficient *para*-cyano aromatic group coupled with a *trans*-alkene linker, resulting in an IC_50_ of 1.7 nM [222]. Inhibitor selectivity studies revealed that compound 17u has 3000-fold higher activity towards NNMT than PNMT. In vitro toxicity assays revealed that compound 17u significantly reduced the viability of HSC-2human oral cancer, A549 human lung carcinoma and T24 bladder cancer cell lines.

Other groups have also designed bisubstrate inhibitors of NNMT. Babault and colleagues developed the bisubstrate mimic MS2734 which has an IC_50_ of 14 µM. However, a methyltransferase screen revealed significant inhibitory activity against DOT1L, PRMT7, BCDIN3D and SMYD2, all proteins involved in epigenetic regulation via histone methylation and miRNA methylation and reported to be dysregulated in cancer [223]. Although not an unwelcome outcome towards tumour cells, the roles of these genes in non-tumour cells may give rise to the possibility of off-target effects. In addition, no screening of its effects upon PNMT and INMT or their efficacy in vitro were carried out [123]. Policarpo and colleagues reported bisubstrate inhibitors with similar efficacies, with methyltransferase screening revealing significant inhibition of INMT, but not PNMT. Toxicity screening of their most potent compound (NS1) revealed no toxicity towards the U2OS human bone sarcoma cell line, which the authors reported was due to poor cell permeability [224]. Chen and colleagues reported the synthesis of bisubstrate inhibitors which utilise a propargyl linkage between the nicotinamide analogue and the adenosine moieties, with one (compound 2a, LL320) having a *k*_i_ of 7 nM. Crucially, specificity screening demonstrated that this molecule exhibited no interaction with either INMT or PNMT, although significant efficacy was observed towards SAH hydrolase (SAHH) [225], essential in the cleavage of SAH and subsequent release of its inhibitory effect upon SAM-utilising enzymes, along with the synthesis of SAM via homocysteine and methionine [226]. Cell permeability screening demonstrated that these molecules did not cross the outer plasma membrane [225].

It must be noted that, in contrast to our studies which used a nicotinamide-based enzyme assay coupled with the direct detection of MNA production using LC–MS, the studies of Babault, Policarpo and Chen all used a SAHH-based fluorescence assay using quinoline as substrate. The use of the SAHH-based fluorescence assay is therefore complicated by the marked inhibition of SAHH by compound 2a, for which it has an IC_50_ of approximately 3 µM [223]. We also reported that NNMT demonstrated only 15% of the catalytic activity towards quinoline compared to nicotinamide, along with possessing substrate inhibition kinetics [63]. What is clear from these studies is that bisubstrate-based inhibitors offer increased efficacy along with increased specificity over and above those of nicotinamide analogues which, due to the nature of the compounds, provide significant scope for further optimisation. One hurdle which clearly needs to be overcome is to enhance the cell permeability of these molecules, using derivatisation with moieties which promote cell permeability. One possibility is to attach moieties which will promote cellular uptake via transporters; however, this raises the very significant possibility of drug–drug interactions [227,228].

### 7.5. Small-Molecule Inhibitors of NNMT Expression

One avenue for targeting NNMT is via its expression. Although, as described previously in this review, several TFBSs have been predicted, only two—STAT3 and HNF-1β—have been functionally confirmed. STAT3 is of particular interest because of its involvement in cancer progression and development of drug resistance. We reported that crispene E, a *cis-*clerodane diterpene isolated from *Tinospora crispa*, inhibited the dimerisation of STAT3 both via an interaction with its SH2 domain and the downregulation of its gene transcription. This correlated with a decrease in the expression of STAT3 target genes, including NNMT. Crispene E had no effect upon the expression of STAT1, thus demonstrating its selectivity. In vitro efficacy and selectivity screening revealed that crispene E was significantly toxic towards the STAT3-positive human breast cancer cell line MDA-MB-231, with an EC_50_ of 5.35 µM, but had no effect against the STAT3-null human leukoblastic leukaemia cell line A4 [159]. A further approach for targeting STAT3 is via its phosophorylation, essential for its transcription factor activity [229,230]. Inhibition of STAT3 phosphorylation using vanillin was shown to inhibit NNMT expression; however, further in vitro and in vivo screening is required to confirm this [122].

YD was shown to supress NNMT mRNA production, which although the exact mechanism was not elucidated, possibly involved in the induction of miR-449a expression [124]. miR-449a is a microRNA whose expression inhibits tumour growth, invasion, and metastasis as well as inducting apoptosis and cellular differentiation [231].

The pleiotropic effects of transcription factors, and the importance of these pathways for non-neoplastic cells, means that they can have wide-ranging effects upon cellular physiology and reduce their cell selectivity. The ability to target NNMT activity using specific inhibitors means that such interventions can be fine-tuned to affect only those pathways affected by enhanced NNMT expression. Additionally, by their very selectivity, off-target effects can be minimised using inhibitors of NNMT activity rather than NNMT expression, making cancer therapeutics much more effective and tolerable for the patient. Therefore, although effective at reducing NNMT expression, the lack of specificity of small-molecule inhibitors of NNMT expression means that it is likely that NNMT inhibitors will be favoured as the most promising therapeutic approach going forward.

## 8. Conclusions

Since the first description of the methylation of nicotinamide in the 1940s [232,233] and its link to SAM usage [234], it was long thought that the role of NNMT was merely as a component of Phase II metabolism, *N*-methylating pyridine-containing compounds and regulating cellular nicotinamide levels [209]. This perception continued until the mid-1980s, after which papers reporting the increased *N*-methylation of nicotinamide in cancer began to be periodically published, albeit at a relatively low rate [130]. The intervening 20 years have seen a rapid increase in interest in the role of NNMT in cancer, fuelled by the increasing knowledge of its involvement in the many cellular pathways essential to both tumour and non-tumour survival. The accumulated evidence of thirty years of research which we have presented in this review clearly shows that increased NNMT expression induces a wide variety of pro-oncogenic effects which serves to support the Warburg effect, to promote tumour metastasis, and to provide the tumour cell with a survival advantage over non-neoplastic cells, a hypothesis which has also been proposed by others [195]. We now have multiple research groups regularly publishing studies of inhibitors with ever-improving binding affinities, inhibitory efficacies, and enzyme selectivity profile. Although unlikely to lead to a cure for cancer when used alone, it is likely that when used as an adjunct therapy NNMT inhibitors will significantly improve the efficacy of other treatments as well as reduce the possibility for the development of drug resistance. It also opens the avenue for the management of cancers for which currently there are limited therapeutic options, for example bladder cancer [235,236]. It is highly likely that, within 5–10 years, we will see NNMT-based therapeutics entering human clinical trials, with the first entering clinical use within 10–15 years.

## Figures and Tables

**Table 1:** Intronic STAT3 and HNF-1β transcription factor binding sites in the *NNMT* gene.

Intron ^a^	Intron Coordinates ^b^	Identified Transcription Factor Binding Motifs	
		STAT3	HNF-1β
2	114,262,935–114,296,427	CTTCCTGGAAT GTTCCTGGAAT CTGCTGGGAAC GTTCTGGAAAA GTGCTAGGAAG CTTTTGGGAAA TTCCTGGGAAA TTCCTGGGAAA GTTCCTGAAAA CTCCTTGGAAA CTGCTAGAAAA	GTCAATTATTTAC TTTAAAAATTAAT GTTATTAATTACC GTAAATCAATTAT TTGAATTATTAAT
4	114,298,160–114,312,044	TTTCTAGGAAT ATTCTGGAAAA GTTCCTGGAAC	TTAAATGATTGAT TTCAATGATTTAT

^a^ Intron number taken from the *NNMT1* transcript. ^b^ Intron coordinates taken from chromosome 11 GRCh38.p13.

**Table 2:** Predicted and experimentally demonstrated post-translational modification sites in the NNMT protein sequence.

Residue	Modification ^a^	Enzyme(s) ^b^	Prediction/Confirmation
Serine-3	Phosphorylation	CK1, GSK3	PhosphoSite [178] (site), Lim et al. [177] (site & enzymes)
Serine-7	Phosphorylation	CK1	Nemmara et al. [179]
Lysine-8	Acetylation	-	PhosphoSite
	Ubiquitinylation	-	
	Methylation	-	
	Succinylation	-	
Tyrosine-11	Phosphorylation	-	PhosphoSite
**Arginine-18**	**Citrullination**	**PAD**	Nemmara et al.
Lysine-23	Ubiquitinylation	-	PhosphoSite
	Succinylation	-	
Tyrosine-24	Phosphorylation	-	PhosphoSite
Tyrosine-25	Phosphorylation	-	PhosphoSite
Lysine-26	Ubiquitinylation	-	PhosphoSite
Serine-29	Phosphorylation	CK1	Lim et al.
Serine-32	Phosphorylation	CK1, PKA2	Lim et al.
Serine-35	Phosphorylation	-	PhosphoSite
Lysine-39	Acetylation	-	PhosphoSite
	Ubiquitinylation	-	PhosphoSite
Lysine-43	Acetylation	-	PhosphoSite
	Ubiquitinylation	-	PhosphoSite
Lysine-47	Ubiquitinylation	-	PhosphoSite
Serine-64	Phosphorylation	CK1	Lim et al.
**Serine-73**	**Phosphoroylation**	**CK2 ^c^**, GSK3	Lim et al.
**Serine-77**	**Phosphorylation**	**CK2 ^c^**	Lim et al.
Lysine-96	Ubiquitinylation	-	PhosphoSite
Lysine-99	Sumoylation	-	Lim et al.
Lysine-100	Ubiquitinylation	-	PhosphoSite
Serine-108	Phosphorylation	GSK3, ProDKin	PhosphoSite (site), Lim et al. (site & enzymes)
Tyrosine-113	Phosphorylation	-	PhosphoSite
Lysine-123	Ubiquitinylation	-	PhosphoSite
**Arginine-132**	**Citrullination**	**PAD**	Nemmara et al.
Lysine-136	Ubiquitinylation	-	PhosphoSite
**Arginine-181**	**Citrullination**	**PAD**	Nemmara et al.
Tyrosine-203	Phosphorylation	-	PhosphoSite
Tyrosine-204	Phosphorylation	-	PhosphoSite
Lysine-210	Ubiquitinylation	-	PhosphoSite
Serine-239	Phosphorylation	GSK3	Lim et al.
Serine-241	Phosphorylation	CK1, GSK3	Lim et al.
Serine-261	Phosphorylation	PKA1	Lim et al.

Residues experimentally demonstrated to undergo post-translational modification are shown in bold. The remaining post-translational modifications are yet to be experimentally confirmed. ^a^ Modifications are colour-coded according to the type of modification. ^b^ CK1 = creatine kinase-1; CK2 = creatine kinase-2; PAD = protein arginine deiminases; GSK3 = glycogen synthase kinase-3; ProDKin = protein-directed kinase-1; PKA1 = protein kinase A1; PKA2 = protein kinase A2; - = not determined. ^c^ The phosphorylation of NNMT by CK2 was demonstrated experimentally using a cell-free assay [177], hence it was not possible to identify which site(s) underwent phosphorylation and whether this was limited to monophosphorylation.

## Data Availability

All novel data reported in this manuscript can be obtained from the authors upon reasonable request.
